# Simulation as a Central Feature of an Elective Course: Does Simulated Bedside Care Impact Learning?

**DOI:** 10.3390/pharmacy6020040

**Published:** 2018-05-03

**Authors:** Michael C. Thomas, Peter J. Hughes

**Affiliations:** McWhorter School of Pharmacy, Samford University, Birmingham, AL 35229, USA; pjhughes@samford.edu

**Keywords:** simulation, emergency medicine, pharmacy practice

## Abstract

A three-credit, simulation-based, emergency medicine elective course was designed and offered to doctor of pharmacy students for two years. The primary objective was to determine if there was a difference in exam performance stratified by student simulation experience, namely either as an active observer or as part of bedside clinical care. The secondary objective was to report student satisfaction. Examination performance for simulation-based questions was compared based on the student role (evaluator versus clinical) using the Student’s *t*-test. Summary responses from Likert scale-based student satisfaction responses were collected. A total of 24 students took the course: 12 in each offering. Performance was similar whether the student was assigned to the evaluation team or the clinical team for all of the comparisons (mid-term and final 2015 and 2016, all *p*-values > 0.05). Students were very satisfied with the course. Of the 19 questions assessing the qualitative aspects of the course, all of the students agreed or strongly agreed to 17 statements, and all of the students were neutral, agreed, or strongly agreed to the remaining two statements. Direct participation and active observation in simulation-based experiences appear to be equally valuable in the learning process, as evidenced by examination performance.

## 1. Introduction

High-fidelity simulation has been used to imitate a behavior or process as part of training in military, aviation, and medical professions [1]. Its use in schools/colleges of pharmacy appears to be common; however, the extent within individual curricula is variable [2]. Many reports detail either a single simulation activity or a limited number of simulation activities [3,4,5,6]. Results from a systematic review of 109 medical simulation studies identified 12 features that lead to effective learning: feedback, repetitive practice, curriculum integration, increasing difficulty, adaptability to various learning styles, a variety of clinical problems, a controlled learning environment, individualized learning, clearly defined outcomes, and the degree of realism [7]. These best practices were included as the framework in the design of an elective course. 

The sole professional pharmacy degree in the Unites States is the doctor of pharmacy (PharmD). Doctor of pharmacy degree programs must be accredited by the Accreditation Council for Pharmacy Education by meeting minimum standards in the professional curriculum [8]. Coursework for PharmD students is commonly completed in four years after students complete their prerequisite coursework. The practice of pharmacy is complex. According to outcomes published by the Center for the Advancement of Pharmacy Education, pharmacists need to be learners, caregivers, managers, promoters, providers, problem solvers, educators, advocates, collaborators, includers, communicators, self-aware, leaders, innovators, and professionals [9]. Similarly, thought leaders were asked to predict the future importance of a number of areas of pharmacy practice. Competencies that were predicted to increase in importance included patient care, using information technology, critical thinking and problem solving, communication, and contributing to patient care teams [10]. Becoming a professional begins with the white coat ceremony for most doctor of pharmacy students in the United States [11]. If pharmacy students are inculcated into the profession after donning the white coat, they should begin to think and act like professionals well before entering their advanced pharmacy practice experiences (APPE). This attitude was summarized in an editorial response written by a student when he called for institutions to provide an environment that helps students grow professionally [12]. However, students need ample opportunity to practice, receive correction, and improve in a safe environment.

Over the last decade, emergency pharmacy practice expanded in the United States, with recent data indicating that 16.4% of hospitals were assigning a pharmacist to the emergency department (ED) [13]. The practice of pharmacy in emergency departments within the United States has been recently described. Based on this nationwide survey of pharmacists in the United States, ED-based pharmacists dedicate a significant amount of time on direct patient care activities related to medication management and time-dependent emergencies such as cardiac arrest, stroke, or trauma [14]. Similarly, opportunities for pharmacy students completing APPEs in this area are offered by most schools/colleges of pharmacy, commonly as electives [15]. However, pharmacy students are unlikely to be exposed to specialized clinical areas such as emergency medicine before their APPEs. With appropriate facilities and expertise, students can experience unique areas of practice in a safe and controlled environment. The present course was purposely designed to blend both didactic and simulation-based learning. This manuscript describes the design of the course, the effect of simulation on examination performance, and student perceptions over a two-year period. This project received institutional review board approval. All of the subjects who took the class opted into the data analysis, and signed an informed consent to be included in the analysis. The main objective of this investigation was to determine if there was a difference in the examination performance for students participating in simulation activities at the bedside versus those who actively observed and evaluated bedside activities. An additional objective was to determine student perception of the course and associated activities using course and supplemental questions. 

## 2. Materials and Methods

**Figure 1:**
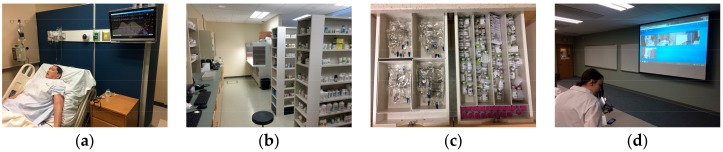
Images showing key elements of the simulated environment: (**a**) Simulation suite showing high-fidelity mannequin; (**b**) Mock pharmacy; (**c**) Simulated medications; (**d**) Classroom showing live video feed of simulation.

The elective course was called Topics in Emergency Medicine, a Simulated Approach. It was designed to give students the opportunity to expand their experiences and practice pharmacy in a simulated hospital setting that included both clinical and operational pharmacy experiences. It was offered and capped at 12 students in the spring semester of the second professional year in a doctor of pharmacy program. Bedside simulation activities were conducted using the simulation suite housed in the college of pharmacy building. The simulation suite contained a high-fidelity mannequin (iStan, CAE Healthcare, Sarasota, FL, USA) and was made to look like a hospital room (Figure 1a). It had a fully functioning hospital bed, emergency cart (simulated medications, airway devices, intravenous fluids, etc.), suction canister, and associated furniture. On a separate floor in the building, there was a mock pharmacy that can be used to simulate retail or hospital pharmacy activities. The mock pharmacy was equipped with a laminar flow hood, multiple computer terminals, and simulated drugs (Figure 1b,c). A third tool that was used for simulation activities was live video streaming technology (LearningSpace, CAE Heatlhcare, Sarasota, FL, USA). Multiple cameras were located in each simulation area, offering several viewpoints. There was the capability to record and live-stream any of the audio and video feeds using any computer with Internet access. Live video technology was used in this course to allow students the ability to evaluate simulation activities in an adjacent room in real time without disrupting the clinical team (Figure 1d).

Both didactic and simulation-based educational strategies were used for each week of this three-credit elective. Topics were chosen by the instructor based on their ability to design simulation activities, and when pharmacotherapy principles were essential for the care of patients in an emergency setting. Each week, a new disease state or condition was covered in a 75-min lecture-based class. After about three weeks of fundamental material, specific clinical content was introduced. Topics included: basic life support, advanced cardiovascular life support, anaphylaxis, electrolyte emergencies, hypertensive emergency, venous thromboembolism, seizures, acute ischemic stroke, acute toxicology, and rapid sequence intubation with post-sedation management. The lectures served as the foundational knowledge that students would need in the simulation experiences. The next 75-min class period was devoted to simulation experiences. At the beginning of the semester, the instructor assigned each student to one of two teams. On simulation days, the class would report to either the mock pharmacy or the simulation area based on their assigned team. Each team had 35 min in each area every week. The teams would switch and report to the other area (mock pharmacy or simulation area).

Although the main focus of the course was related to emergency pharmacy practice, students were also given the opportunity to experience the operational aspects of a mock hospital pharmacy. When the course was designed, the instructor wanted learners in the course to experience a full sense of realism whenever possible. Using the mock pharmacy allowed expanded realism, decreased group size in the simulation suite, and allowed the practice of previously learned skills such as making parenteral compounds. For example, if a medication was needed in the simulation suite, a verbal order would need to be called in to the inpatient mock pharmacy, and subsequently the medication would be prepared, checked, and delivered to the bedside. These steps of the medication use processes are important for pharmacy practice in the hospital environment. They allowed students in the mock pharmacy to exercise practical skills while increasing a sense of realism. As students reported to their assigned area, they would learn their assigned role for the day. There were six defined roles in the mock pharmacy. These were designed so that students would have a variety of rotating experiences. Each role had an associated letter labeled: A, a, B, b, C, c. The roles and responsibilities were reviewed prior to the first simulation experience, and the definitions of each role were included on printed instructions located in the mock pharmacy. The responsibility of each role was as follows: ‘A’ received telephone orders, entered the orders into the pharmacy order entry system (Neehr Perfect), and answered the drug information question. The role of ‘a’ was to help the ‘A’ with the drug information question, verify the appropriate entry of the medication order, and print any labels that were needed for the telephone order. There were two roles (B, b) focused on parenteral medications. One was responsible for cleaning the laminar flow hood, and they were responsible for preparation of parenteral products. The other role was responsible for checking a pre-arranged intravenous (IV) batch. This was typically five to six products that contained two to three planted mistakes. Any mistakes identified required the student making parenteral products to prepare the correct product. The final two roles (C, c) were focused on the entry and verification of admission orders. Printed orders were provided, and medication orders were entered into the pharmacy order entry system with subsequent verification. All six roles were intentionally designed to provide students with practical experiences in inpatient pharmacy environments. Since the course had multi-week simulations, each student had experience in each role.

**Figure 2:**
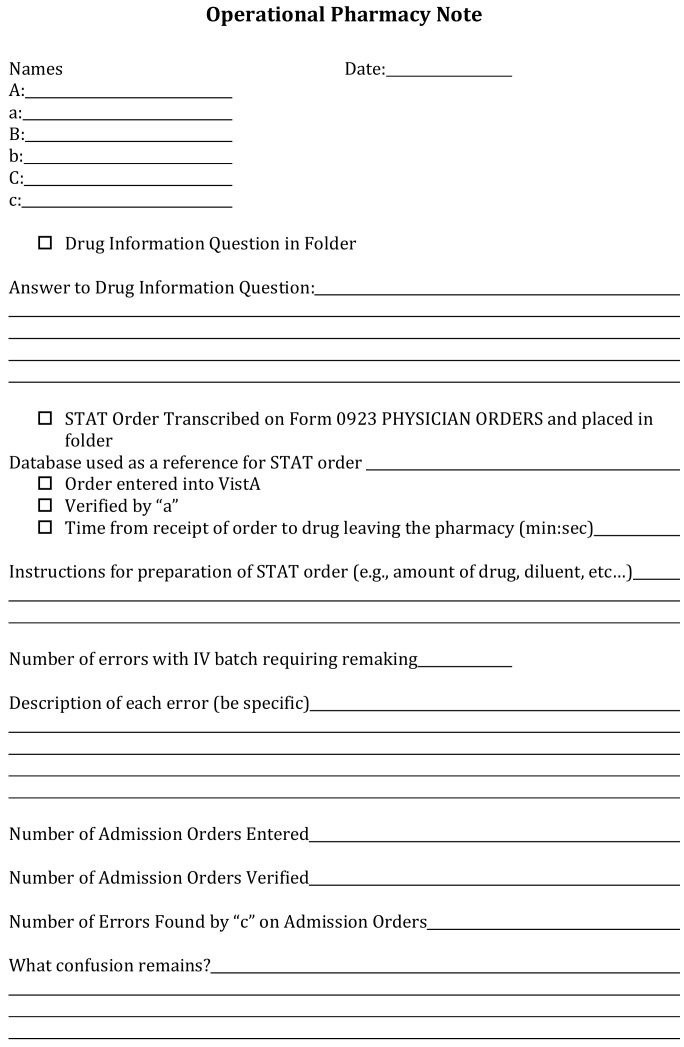
Operational pharmacy note template.

The teams in the mock pharmacy use operational pharmacy notes to track the completion of their tasks (Figure 2). This template is where they documented the answer to their drug information question, how many errors they found in the IV batch, the results of their peer-to-peer order entry, and a space to communicate any issues that may still be confusing to members of the team. 

**Figure 3:**
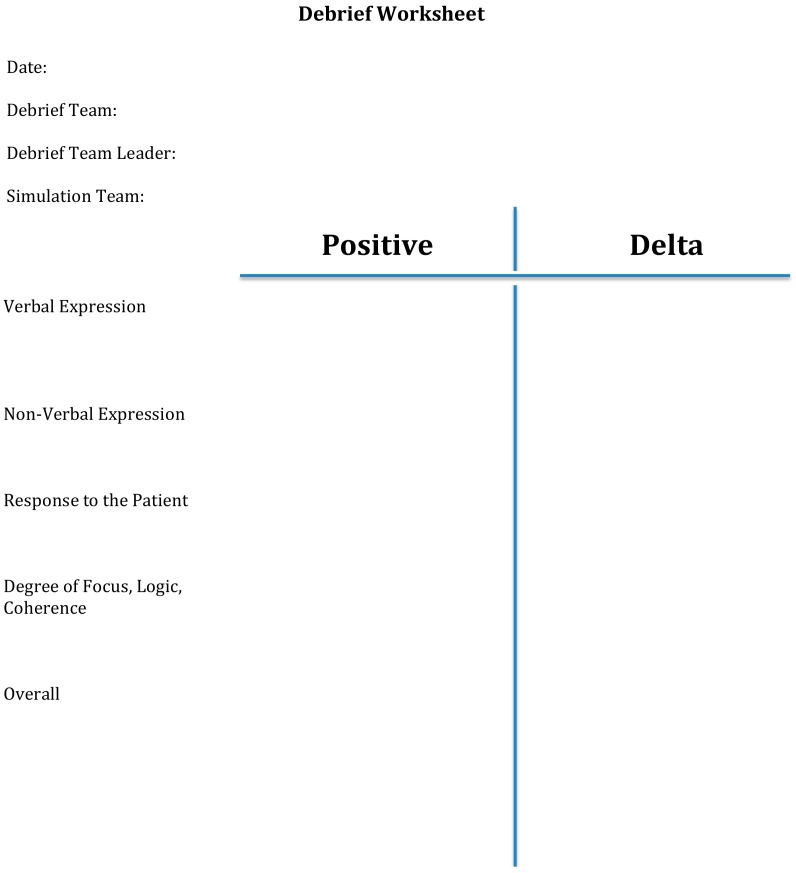
Debrief worksheet.

The other team was further split into two groups of three, and reported to the simulation area. One group entered a classroom adjacent to the simulation suite, and was responsible for evaluating and debriefing the clinical team that was taking care of the patient. If students were on the clinical team one week, they would be on the evaluation team the following week, and vice versa. This allowed an equal distribution of experiences. They had several camera views projected onto a large screen of the team interacting with the high-fidelity mannequin in addition to a feed that showed the vital signs and waveforms (e.g., an electrocardiogram lead) of the mannequin. The debrief team used a form that was designed for this class to guide the debrief of the clinical team on five domains (verbal expression, non-verbal expression, response to the patient, degree of focus, logic, coherence, and overall performance), and provide feedback on areas of strength (+) and areas of improvement (delta) for each domain (Figure 3). 

The three students who were assigned to the simulation suite had limited or no information about the patient before entering the room. However, students were aware that the main problem would be from the previous class’s didactic material. When they entered the room, they needed to gather information from multiple sources. Information may come from an actor playing the part of a family member in the room, the patient, the medical chart, or a combination of these elements. During the 15-min simulation, students needed to synthesize the important information that was necessary to make decisions (focused physical exam, allergies, home medications, etc.). If the patient required immediate treatment, including pharmacotherapy, it was the students’ responsibility to order it from the mock pharmacy (if not in the crash cart) or administer it to the patient (if it was in the crash cart) with appropriate reassessment. At the end of 15 min, the simulation was terminated, and the clinical team had 15 min to compose a clinical progress note. During this time, the debrief team (those watching from the adjacent room) also used this time to discuss their observations and complete the debrief form. After 15 min, the debrief team entered the simulation suite and guided a discussion about the team’s performance, including positive elements and opportunities for improvement. Similar to the mock pharmacy experience, the roles of students rotated from week to week, and each student had multiple experiences with each role throughout the semester.

During simulation days, the main role of the instructor was facilitator. Due to the time-sensitive nature of each activity, the instructor would provide time cues or delegate time cues as appropriate. However, every effort was made to avoid entering the simulation room until the time was up with the high-fidelity mannequin in order to preserve authenticity. The instructor would stop into the mock pharmacy and ensure there were not any questions or technology issues. Oftentimes, students would ask about procedural issues that they may face as a pharmacist in a hospital setting. While the debrief team watched the simulation, the instructor could probe the small group for questions on how the simulation group was doing with communication, therapeutic decision making, acting on critical vital signs, or similar lines of questioning. All of the simulation events were recorded, archived, and available to the instructor for review. 

## 3. Results

This investigation focused on two areas of assessment. The first was performance on examination questions stratified by student role in the simulation (evaluation team or clinical team), and the second was student satisfaction with the course. 

Performance in this course was evaluated through assignment completion, participation, and examination performance. A total of 55% of the course grade was group performance, and 45% was based on individual efforts. Group efforts were graded through assessing clinical (25%) and operational pharmacy notes (25%) against a grading rubric. Additionally, groups were assessed on their debrief note and discussion with the clinical team (5%) Individually, students were assessed for professionalism and participation (10%) and performance on two written examinations, a mid-term (17.5%) and comprehensive final examination (17.5%). The examinations were paper-based and included fill-in-the-blank, multiple choice, and matching. The questions covered material from simulations and the didactic portion of the course. 

Twenty-four students were enrolled in the course: 12 in each semester it was offered. One student in the second offering of the course was unable to finish, but did complete the mid-term examination and associated course activities. 

The course design allowed students to equally experience simulation at the bedside and being part of the debrief team. Examination questions were written based on learning objectives for the didactic portion of the course. Some learning objectives were reinforced during the bedside simulation activities. Content on examinations could be derived solely from the didactic lecture or, when it was also experienced, in the simulation suite. Using the simulation objectives, the instructor determined whether every question on mid-term and final examinations addressed experiences in the simulation. Since students were either on the clinical team or evaluation team for every simulation, this allowed for a comparison of exam performance on questions directly addressed by their role in the simulation experiences (Table 1). Using the Student’s *t*-test, clinical and evaluation teams performed similarly on mid-term and final examinations over two years. The amount of each examination that was directly attributed to simulation objectives was: 25 out of 50 (50%) for the mid-term in 2015; 57 out of 77 (74%) for the final in 2015; 19 out of 52 (37%) for the mid-term in 2016; and 37 out of 77 (48%) for the final in 2016. 

Feedback on the class was sought from students using a standard course evaluation instrument and a supplementary set of questions specific to this course. These evaluations were administered during the last week of each semester according to guidelines established at the college of pharmacy. Each question or domain was assessed on a five-point Likert scale (Strongly Disagree, Agree, Neither Agree or Disagree, Agree, Strongly Agree). Students were also free to submit open-ended comments. 

Students rated a total of 21 evaluative statements. The course was very well received. All of the students agreed or strongly agreed to 19 of the statements, and 95% agreed or strongly agreed to the two remaining statements. No statement received a score of less than neutral. The complete results are shown in Table 2. The course evaluation instrument generated 71 total comments. The majority of comments were positive (*n* = 68; 94%), and were categorized into seven categories: Enjoyable (*n* = 23), Challenging (*n* = 10), Effective/Beneficial (*n* = 10), Teamwork (*n* = 2), Realism (*n* = 8), and Learning (*n* = 15). The comments that were related to improvement opportunities included two that felt increased time for simulation would be beneficial, and one desired a different pharmacy order entry system.

## 4. Discussion

In this novel simulation-based emergency medicine elective course, performance on objective examination questions did not differ based on participation in bedside simulation activities on the clinical team or active observation through participation on the evaluation team. Additionally, the course was well received, and students felt that the experiences prepared them for authentic patient care activities. This course is unique in educating pharmacy learners in emergency medicine because it provided repeat simulation experiences over the entire semester. This repetitive element is a core feature that leads to effective learning using simulation [7]. Only one other example was found in the pharmacy education literature of repetitive simulation activities [16].

Students were exposed to eight to 10 simulation activities. For about half of the activities, students were active observers of the clinical team in the simulation suite, and for the other half of the activities, the students were active participants as part of the clinical team. Simulation-based learning activities allow students to practice taking care of patients in safe environments where mistakes become teachable moments instead of medical errors. Simulation-based activities help students practice skills such as communication and physical assessment while integrating clinical aspects of care. 

There is an ongoing debate as to the “practice readiness” of doctor of pharmacy graduates [17,18]. It likely depends on the area of pharmacy practice; however, students learn more through active learning than passive methods [19]. Active learning requires an application of knowledge and skills, and helps students apply information to future situations [20]. Few studies have been conducted to compare simulation-based activities to traditional instruction. However, one study of simulation-based activities was shown to be superior to problem-based learning in a group of fourth-year medical students [21]. Although the design of this course did not allow the measurement of distinct methods of teaching, it did allow for a comparison based on experiences within the course (clinical team versus evaluation team). The results showed that during simulation experiences, active observation or participation produced a similar performance on examination questions. It should be noted that all of the students received the same didactic lecture material, and all of the exam questions were directed toward the attainment of didactic learning objectives. By virtue of the course design, some of these objectives were reinforced during simulation-based activities. The results are reassuring, because taking part in both teams (observer or participant) was deemed important to the design of the course. In other investigations, it was not clear whether simulation improved examination performance. For example, a recent study of doctor of pharmacy students showed that performance on a written examination was no different if students took part in classroom lecture or a high-fidelity simulation for teaching advanced cardiac life support [20]. Other studies have shown improvement in pre-knowledge and post-knowledge [4,16,22]. 

In regards to student perceptions of the course, the results were very positive, and are consistent with other reports of student perceptions of learning and the effectiveness of simulation-based activities [3,6,11,23,24]. All of the students agreed or strongly agreed with the statement: “This course helped me develop skills I can use on APPE rotations in hospital settings”. Similarly, all of the students selected agree or strongly agree to the statement: “Considering the content covered in the course, I would be able to positively contribute to the care of a real patient”. Both of these lend support for a step toward practice readiness. 

This course provided weekly opportunities for students to apply material learned in the classroom and test their understanding in settings that they will encounter in a hospital-based practice. They were challenged to provide formative feedback to their peers and work under tight time constraints to get all of the work done. Over the semester, they became more efficient and adept at each of the assigned tasks. Observationally, they seemed to treat the mannequin as a real patient. Students conducted themselves in a professional manner while at the bedside. One element that was likely underestimated at the genesis of the course was the importance of communication as a central feature. Over time, the teams improved their ability to gather information from the patient, the chart, and the patient monitor. Students improved internal communication, and worked more efficiently and effectively as a team. The clinical team received both praise and criticism from the debrief team, which shaped and improved communication and decision making in the simulation suite. The debrief teams demonstrated interpersonal communication skills as they filled out the debrief worksheet, and decided what elements to include. Finally, they communicated their findings with respect. Students actively watching the simulation had the luxury of talking without disrupting the simulation. It also afforded opportunities for the instructor to capitalize on teachable moments while in the room observing the simulation. 

Although not directly assessed on examinations, the experiences in the mock pharmacy provided realistic experiences. The operational pharmacy teams rotated their roles, and each student had experiences that were similar to an inpatient hospital pharmacy. They worked to support the care of the patient in the simulation suite by taking a telephone order, properly labeling the product, and delivering it to the bedside in a short amount of time. The focus during each simulation experience was less about the right decision, and more about the process and learning from mistakes. When common misconceptions arose, these were reviewed during the didactic class time.

The qualities represented in this course are especially important since the publication and endorsement of the core entrustable professional activities (EPAs) for new pharmacy graduates [25]. The explicit nature of these activities is to set a baseline or standard for performance tasks that new graduates should be able to complete upon graduation from a doctor of pharmacy program. Specifically, the activities utilized in this elective course satisfied five of the six EPA domains (i.e., patient care provider domain, population health promoter domain, information master domain, and practice manager domain) [25]. Activities were not built into this course to satisfy the interprofessional team member domain, although this domain is a logical next step for the evolution of a course of this nature. Undergirding all of the core EPA domains are the tenets of professionalism, self-awareness, and communication, which are all skills or traits upon which students enrolled in this elective course depended for successful completion [25]. 

An essential aspect of this course was the use of a high-fidelity simulation mannequin. While this may be a perceived barrier, many colleges and schools have access to this technology. A survey conducted in 2013 reported that of the 88 responding schools, 30 had their own high-fidelity mannequin, and 47 had access to a formal simulation center [2]. For any simulation experience to be effective, sufficient planning is necessary to develop a sense of realism. Cases must be well designed knowing the capabilities of the equipment and personnel. For this course, cases were developed by a single instructor and reviewed with the simulation manager before each simulation activity. The simulation manager served as the voice of the patient and controlled physiologic responses as the disease was treated or not treated by students during the simulation. The instructor for the course could communicate with the simulation manager during the case using text messaging if there were obvious mistakes requiring dynamic adjustments to the case. For example, if students failed to provide epinephrine to a patient with anaphylaxis in a timely fashion, the patient’s clinical condition would be programmed to deteriorate. Over the semester, cases became more complex, and required a chart with some elements at the bedside. For example, a 12-lead electrocardiogram was printed and put at the bedside for the pulmonary embolism case. Other times, there would be a focused physical exam note by the emergency physician noting his or her findings. This was because some things cannot be easily simulated, such as the results of a fundoscopic exam, or findings from the skin such as tenting, dry mucous membranes, or diaphoresis. When lab results were important, these were also placed on the bedside chart. A patient name band was also created using standard address labels affixed to colored paper and wrapped around the wrist. It contained the name of the patient and their date of birth.

To increase realism in the operational pharmacy, basic supplies were purchased. These included syringes, needles, alcohol pads, intravenous piggyback solutions in various sizes, and cleaning supplies for the laminar flow hood. Fortunately, there was a working laminar flow hood where parenteral products could be made in the mock pharmacy. This helped to increase realism; however, the activity could also be done on a workbench, and a flat surface could be defined as the laminar flow hood. Since simulated drugs can be expensive, every attempt was made to reuse supplies. For example, empty vials were used for the batch, and base solutions were continuously reused. Standard address labels containing drug names and concentrations were affixed to 10 mL of sterile water or 0.9% sodium chloride vials so that virtually any drug could be made available. Each week, about six to eight vials were used with the associated syringes and needles. To maintain realism, when compounding parenteral products, students would only use new vials, needles, and syringes.

There are several limitations to this investigation. First, there was some heterogeneity with respect to content delivery and testing from the first to second year. This was based on continuous improvement efforts and course refinement. However, the framework of the course remained unchanged, it was taught by a single instructor, and data analysis was conducted based on the experiences of each cohort. Secondly, the number of students was relatively small, and the investigation was conducted at a single institution. 

It should be noted that implementing a course such as this could be very time consuming, especially during the planning phases. If details are not worked out, realism soon fades, and student confidence in the design may suffer. Checklists worked well to mitigate this potential challenge. 

## 5. Conclusions

Performance on objective examination questions did not differ based on participation in bedside simulation activities or evaluating the clinical team. The course was very well received, and students felt that it prepared them for APPE experiences in the hospital setting, and to ultimately take care of patients. 

## Figures and Tables

**Table 1:** Average performance on examination questions linked to simulation objectives.

Examination	Clinical Team (Mean ± SD)	Evaluation Team (Mean ± SD)	*p*-Value
Mid-term 2015 (*n* = 12)	67.5 ± 13.5%	71.6 ± 12.1%	0.30
Final 2015 (*n* = 12)	84.6 ± 6.3%	82.0 ± 6.2%	0.24
Mid-term 2016 (*n* = 12)	76.6 ± 11.5%	68.4 ± 12.9%	0.12
Final 2016 (*n* = 11)	72.7 ± 7.6%	73.2 ± 5.2%	0.45

SD = standard deviation.

**Table 2:** Course evaluation responses by students.

Statement	SD %	D %	N %	A %	SA %
The course objectives were well covered (*n* = 22)	0	0	0	9.1	90.9
The course expectations were met (*n* = 22)	0	0	0	9.1	90.9
The course challenged me intellectually (*n* = 22)	0	0	0	4.5	95.5
The course concepts were presented in an organized manner (*n* = 22)	0	0	0	13.6	86.4
Instructional material(s) increased my understanding (*n* = 22)	0	0	0	4.5	95.5
The course assignments were interesting and stimulating (*n* = 22)	0	0	0	4.5	95.5
The course helped me to develop stronger critical thinking skills (*n* = 22)	0	0	4.5	9.1	86.4
This course helped me develop skills I can use on APPE rotations in hospital settings (*n* = 20)	0	0	0	5	95
The simulation days helped me apply what I learned in the classroom (*n* = 20)	0	0	0	10	90
Bedside simulation using the high-fidelity simulation man (iStan) was valuable to my learning (*n* = 20)	0	0	0	5	95
Order entry in the mock pharmacy was valuable to my learning (*n* = 20)	0	0	5	30	65
Making intravenous admixtures was valuable to my learning (*n* = 20)	0	0	0	10	90
Checking order entry was valuable to my learning (*n* = 20)	0	0	0	20	80
Checking intravenous admixtures was valuable to my learning (*n* = 20)	0	0	0	20	80
Receiving and transcribing a telephone order was valuable to my learning (*n* = 20)	0	0	0	20	80
The course improved my ability to self-assess (*n* = 20)	0	0	0	25	75
The course improved my confidence in making intravenous admixtures (*n* = 20)	0	0	0	25	75
The course improved my communication skills amongst team members (*n* = 20)	0	0	0	20	80
The course improved my ability to effectively communicate with patients in the acute care setting (*n* = 20)	0	0	0	25	75
The simulation and mock pharmacy experiences felt realistic	0	0	0	15	85
Considering the content covered in the course, I would be able to positively contribute to the care of a real patient	0	0	0	15	85

SD = Strongly disagree; D = disagree; N = Neutral; A = Agree; SA = Strongly Agree; APPE: advanced pharmacy practice experiences.
